# Vitamin D3 enhances the tumouricidal effects of 5-Fluorouracil through multipathway mechanisms in azoxymethane rat model of colon cancer

**DOI:** 10.1186/s13046-015-0187-9

**Published:** 2015-07-25

**Authors:** Bassem Refaat, Adel Galal El-Shemi, Osama Adnan Kensara, Amr Mohamed Mohamed, Shakir Idris, Jawwad Ahmad, Athar Khojah

**Affiliations:** Laboratory Medicine Department, Faculty of Applied Medical Sciences, Umm Al-Qura University, Al-Abdeyah, PO Box 7607, Makkah, Kingdom of Saudi Arabia; Department of Pharmacology, Faculty of Medicine, Assiut University, Assiut, Egypt; Clinical Nutrition Department, Faculty of Applied Medical Sciences, Umm Al-Qura University, Al-Abdeyah, PO Box 7607, Makkah, Kingdom of Saudi Arabia; Clinical Laboratory Diagnosis, Department of Animal Medicine, Faculty of Veterinary Medicine, Assiut University, Assiut, 71526 Egypt

**Keywords:** Colon cancer, Vitamin D3, β-catenin, TGF-β, iNOS, HSP-90, COX-2, Therapeutic efficacy

## Abstract

**Background:**

Vitamin D3 and its analogues have recently been shown to enhance the anti-tumour effects of 5- Fluorouracil (5-FU) both in vitro and in xenograft mouse model of colon cancer. This study measured the potential mechanism(s) by which vitamin D3 could synergise the tumouricidal activities of 5-FU in azoxymethane (AOM) rat model of colon cancer.

**Methods:**

Seventy-five male Wistar rats were divided equally into 5 groups: Control, AOM, AOM-treated by 5-FU (5-FU), AOM-treated by vitamin D3 (VitD3), and AOM-treated by 5-FU + vitamin D3 (5-FU/D). The study duration was 15 weeks. AOM was injected subcutaneously for 2 weeks (15 mg/kg/week). 5-FU was injected intraperitoneally in the 9th and 10th weeks post AOM (8 total injections were given: 12 mg/kg/day for 4 successive days, then 6 mg/kg every other day for another 4 doses) and oral vitamin D3 (500 IU/rat/day; 3 days/week) was given from week 7 post AOM till the last week of the study. The colons were collected following euthanasia for gross and histopathological examination. The expression of β-catenin, transforming growth factor-β1 (TGF-β1), TGF-β type 2 receptor (TGF-βR2), smad4, inducible nitric oxide synthase (iNOS), and heat shock protein-90 (HSP-90) proteins was measured by immunohistochemistry. In colonic tissue homogenates, quantitative RT-PCR was used to measure the mRNA expression of Wnt, β-catenin, Dickkopf-1 (DKK-1) and cyclooxygenase-2 (COX-2) genes, while ELISA was used to measure the concentrations of TGF-β1, HSP-90 and COX-2 proteins.

**Results:**

Monotherapy with 5-FU or vitamin D3 significantly decreased the number of grown tumours induced by AOM (P < 0.05); however, their combination resulted in more significant tumouricidal effects (P < 0.05) compared with monotherapy groups. Mechanistically, vitamin D3/5-FU co-therapy significantly decreased the expression of Wnt, β-catenin, iNOS, COX-2 and HSP-90 and significantly increased the expression of DKK-1, TGF-β1, TGF-βR2, smad4 (P < 0.05), in comparison with their corresponding monotherapy groups.

**Conclusions:**

Vitamin D3 and 5-FU synergise together and exhibit better anticancer effects by modulating Wnt/β-catenin pathway, TGF-β1 signals, iNOS, COX-2 and HSP-90. Further studies are required to illustrate the clinical value of vitamin D supplementation during the treatment of colon cancer with 5-FU in human patients.

## Background

Colorectal cancer (CRC) is the third most common malignancy and is ranked as the fourth leading cause of deaths from cancer worldwide according to the World Health Organization [1]. Azoxymethane (AOM) is commonly used in rodents to create an experimental model of colon cancer that mimics the sporadic phenotype in human with high similarities in molecular, clinical and histopathological features of the disease between both species [2–4]. AOM has therefore been extensively used in the study of the molecular biology, prevention and treatment of CRC [2, 4]. Metabolites of AOM cause mutagenesis of colon glandular epithelial cells DNA by replacing the nucleotides from G:C to A:T and the required duration for the development of AOM-induced colon cancer is 14 weeks in rodents [2, 4, 5].

Pathological alterations in several molecular pathways have been identified in the tumorigenesis of colon cancer. These molecules include transforming growth factor (TGF)-β and its related molecules, Wnt/β-catenin pathway, inducible nitric oxide synthase (iNOS), heat shock protein (HSP)-90 and cyclooxygenase (COX)-2 [2]. TGF-β inhibits the growth of normal epithelial cells and it has been found that cancer cells become resistant to the growth inhibitory effect of the molecule. Possible mechanisms of resistance include a decrease in the expression of the protein, its type 2 receptor and mutation of its intracellular mediators, smad2 and 4, in both human and rodent model of colon cancer [6–8]. Another major pathogenic mechanism of CRC is based on the abnormal increase in the expression of Wnt molecule that results in the stabilization of β-catenin and its localization in the nucleus. This effect is inhibited by *Dickkopf* (DKK) family and DKK-1 is the most extensively studied member of the family [9–11].

Other studies have also demonstrated that iNOS and COX-2 play a crucial role in the development of colon cancer in human and AOM-induced model in rodents as both are pro-angiogenic molecules and they promote tumour angiogenesis, which is a vital step for the progression and spread of solid tumours [12–14]. Furthermore, The use of iNOS inhibitors and non-steroidal anti-inflammatory drugs, such as COX-2 inhibitors, have shown to exhibit antitumor properties by several studies [14, 15]. HSP-90 is another molecule involved in the pathogenic mechanisms of colon cancer. HSP-90 regulates protein folding and trafficking in normal cells and blocking the activities of HSP-90 has been shown to delay the progression of colon cancer in vitro and in vivo by several mechanisms [16–18].

Surgical removal of colon cancer is the most effective therapeutic approach but it requires early diagnosis [19]. In advanced stages of the disease, chemotherapy and radiotherapy are other alternative approaches [20, 21]. One of the most common chemotherapeutic agents used for the treatment of CRC is 5-Fluorouracil (5-FU) [20–22]. However, the success of achieving significant suppression of cancer by chemotherapy is rare and the 5 years survival rate for metastatic colon cancer is < 10 % [20, 21, 23, 24]. Hence, understating the cancer biology and its related pathophysiological mechanisms is necessary for the development of effective therapeutic strategies. A possible approach to achieve such development is the combination of other agents that could enhance the therapeutic effect of 5-FU.

Many studies have proposed vitamin D and calcium as effective chemopreventive agents against the development of CRC in human and chemically induced rodent models [25–28]. Vitamin D is also involved in the regulation of cell proliferation, differentiation, apoptosis, cell cycle and angiogenesis [22, 29]. Several other reports have also shown positive effects of combining active vitamin D or its analogues with a variety of chemotherapeutic agents in the treatment of different cancers [30–32]. Recently, supplementation with vitamin D analogues has been shown to increase the sensitivity of colon cancer cells to 5-FU and to enhance the cytotoxic effects of the drug both in vitro and in vivo [22, 33–35].

These aforementioned findings suggest that combining vitamin D and 5-FU may be promising in the treatment of CRC. The current study therefore measured the effects of combining vitamin D3 with 5-FU on colon cancer regression and the expression of Wnt/β-catenin, TGF-β1 and its related molecules, HSP-90, iNOS and COX-2 in an intermediate (15 weeks) AOM experimental model in rat. A better understanding of the mechanisms by which vitamin D enhances the cytotoxic efficacy of 5-FU may provide a better therapeutic approach for the treatment of colon cancer, especially in those patients with advanced stages of the disease.

## Methods

### Drugs

AOM and 5-FU were purchased from Sigma-Aldrich (St. Louis, MO, USA). Vitamin D3 (cholecalciferol 4500 IU/mL) oral drops (VitD3, Novartis International AG, Basel, Switzerland) was used in the study.

### Study design

All experimental protocols were approved by the Committee for the Care and Use of Laboratory Animals at Umm Al-Qura University and were in accordance with the EU Directive 2010/63/EU for animal experiments.

A total of 75 male Wistar rats weighing 200–250 gm were used. All animals received humane care during the study protocol and during euthanasia. The animals were housed in clean and sterile polyvinyl cages (5 rats/cage), maintained on standard laboratory pellet diet and water *ad libitum*; and kept in a temperature-controlled air-conditioned at 22–24 °C and 12 h dark/light cycle. After acclimation, the rats were randomly categorised into 5 groups (15 rats/group) as follows: The first group served as ‘Control group’, the second group consisted of those that only received AOM ‘AOM group’, the third group received AOM + 5-FU ‘5-FU group’, The fourth group received AOM + vitamin D ‘VitD group’ and the last group consisted of those that received AOM + 5-FU + VitD ‘5-FU/D group’.

### Treatment protocol

AOM was dissolved in normal saline and injected subcutaneously to the animals at a dose of 15 mg/kg body weight, once weekly for a total of 2 weeks to induce tumorigenesis in the colon as previously described [24]. 5-FU was dissolved in normal saline and injected intraperitoneally to the designated groups during the 9th and 10th weeks of the study in a dosage regimen similar to that used for the treatment of colon cancer in human (8 total injections were given: 12 mg/kg/day for 4 successive days, then 6 mg/kg every other day for another 4 doses). The drugs were prepared fresh on the day of use.

Cholecalciferol (4500 IU/mL) was prepared by adding 4 ml to 16 ml saline every morning to form a final concentration of 1000 IU/mL. Each rat in the ‘VitD’ and ‘5-FU/D’ groups received 0.5 ml/day (500 IU/day; 3 days/week) by oral gavage from week 7 post AOM till the last week of the study protocol. Cholecalciferol and its dose were chosen over calcitriol, the hormonal form of vitamin D, to avoid the risk of soft tissue calcification [36].

### Types of samples

Rats were fasted overnight and subsequently euthanised under anaesthesia using diethyl ether (Fisher Scientific UK Ltd, Loughborough, UK). Three ml of blood were collected from each rat in a plain tube immediately after cutting the vena cava. The samples were centrifuged and the serum was stored in −20 °C till used to measure the serum levels of liver enzymes (ALP, ALT and AST), renal function parameters (creatinine, BUN and urea) and serum concentrations of 25-OH vitamin D using Cobas e411 (Roche Diagnostics International Ltd, Switzerland) according to the manufacturer’s protocol.

Whole colon from rectum to caecum was gently resected, flushed with cold potassium phosphate buffer (0.1 M, pH 7.2) to remove residual bowel contents, slit opened longitudinally, its length was measured, and then submerged overnight in 10 % (v/v) neutralised formalin with the mucosa on the upper side between layers of filter papers. The length and width of the isolated colons were measured to calculate the colon surface area as follow: Length X Width in cm^2^. All colon specimens were then processed for gross and histopathological examination and later for immunohistochemistry, ELISA and quantitative gene expression.

### Gross and microscopic quantification of tumours

The grown tumours on colon mucosa were counted by 2 blinded observers to the source group by naked eye. Next, the formalin-fixed tissues were cut into proximal, middle and distal segments of the same length. Each segment was stained with 0.2 % methylene blue solution for 1.5–2 min, placed on a microscope slide with the mucosal side upward, and then observed under a dissecting microscope to examine and count the numbers of small tumours that were not detected by naked eye. All segments of each colon were also examined by two observers who were blind to the source group.

A micro-feather scalpel blade was used under the dissecting microscopy to excise the tumours of interest from the surrounding normal tissues to be used for histopathological, immunohistochemical and molecular examinations. Two specimens were processed for histopathology, 2 specimens for immunohistochemistry and the remaining were distributed equally either in RIPA buffer (Santa-Cruz Biotechnology Inc, Burlingame, CA) for protein extraction or RNA*Later* (Ambion, Thermo Fisher Scientific, USA) for preservation in −80 °C till processed for quantitative RT-PCR.

### Histopathological examination

Following de-staining from methylene blue with 80 % ethanol, the tissue specimens were processed by a conventional method, embedded in paraffin and sectioned at 4–5 μm, and stained by haematoxylin and eosin (H&E) for histopathology. Aberrant crypt foci (ACF) and glandular epithelial morphology were examined by an expert histopathologist who was blind to the specimen group. Based on the crypt architecture and nuclear features, ACF were microscopically classified into hyperplastic ACF (no dysplasia) or dysplastic ACF (elongated, crowded and pseudo-stratified nuclei; increased nucleus-to-cytoplasm ratio; reduced number of goblet cells; back-to-back glands and markedly decreased interglandular stroma) similarly to the previously established and published criteria [37]. A colonic adenoma consisted of proliferative/hyperplastic colonic glands, while a colonic adenocarcinoma was characterised by dysplastic glands that invaded the submucosal muscle layer [4].

### Immunohistochemistry

Polyclonal goat IgG antibodies to detect TGF-β1 (C-16), smad4 (C-20), β-catenin (C-18), and polyclonal rabbit IgG antibodies against TGF-β type II receptor (L-21), iNOS (N-20) and HSP-90-α/β (N-17) were obtained from Santa-Cruz Biotechnology Inc (Burlingame, CA).

An avidin-biotin horseradish peroxidase technique was used to localise the proteins of interest. Briefly, paraffin embedded sections of 5 μm thickness were dewaxed in xylene, dehydrated in alcohol, and treated with 2 % (vol/vol) hydrogen peroxide for 20 minutes in methanol to block endogenous peroxidase. All sections were pre-treated in an 850-watt domestic microwave oven in 0.01 M citrate buffer for 3 minutes. The sections were incubated for 30 minutes with normal donkey or normal goat serum for primary goat and rabbit IgG antibodies, respectively. The sections were then incubated with the primary antibodies (the antibody concentration was 1:100 for all used antibodies) over night at 4 °C.

The following day the sections were washed with 20 mM PBS (pH 7.3) and then incubated with 1:200 biotinylated anti-goat or anti-rabbit secondary antibodies for 30 minutes. After a further wash step, the sections were incubated with the avidin-biotin peroxidase complex ABC system (Santa-Cruz Biotechnology Inc, Burlingame, CA) for 30 minutes and then subsequently with 3, 3′-diaminobenzidine (Santa-Cruz Biotechnology Inc, Burlingame, CA) for 10 minutes. Sections were washed in tap water, counter stained with Gill’s haematoxylin, then dehydrated in a series of graded ethanol, cleared in xylene, and mounted in DPX (BDH/Merck, Leicestershire, UK). The negative control slides consisted of a section of the tissue block being studied, which was treated identically to all other slides, with the exception that the primary antibody was omitted to control for non-specific binding of the detection system.

For evaluation and scoring of immunohistochemical staining, the sections were observed on a Labor Lux microscope (Leitz, Wetzlar, Germany), at a magnification of ×100, ×200 and ×400. A positive reaction was characterised by the presence of brown staining. Each section was examined by two observers who were blinded to the source of tissue and the intensity of staining was assessed using the ‘H score’ which was calculated using the following formula [38]: H score = ∑P_ί_ (ί +1), where ί represents the intensity of staining (0 = negative; 1 = weak; 2 = moderate and 3 = strong) and P_ί_ is the percentage of cells (0–100 %) stained at each intensity.

In the case of a wide disagreement between the two observers, the slides were reanalysed by a third independent reviewer. The final result was obtained by averaging the individual observer results. Representative sections were photographed using an Olympus digital camera at ×200 magnification.

### Enzyme linked immunosorbant assay (ELISA)

Two specimens measuring 1 cm each that involved tumours (except for the control group) were obtained from each colon following excision under dissecting microscope and they were used immediately for protein extraction using 2 ml of RIPA lysis buffer containing protease inhibitors (Santa-Cruz Biotechnology Inc, Burlingame, CA) and electrical homogeniser. All homogenated samples were centrifuged at 14000 rpm for 30 minutes and small aliquots (0.5 ml) of the resultant supernatant were placed in Eppendorf tubes and stored in −20 °C till processed to measure the levels of candidate proteins in colon using ELISA.

The concentrations of total proteins extracted from the colon tissue homogenates were measured using the BioSpec-nano (Shimadzu Corporation, Japan) at 280 OD. All protein samples were diluted using normal sterile saline to make a final concentration of 500 μg/ml of total protein.

The concentrations of TGF-β1, HSP-90α proteins and COX-2 enzyme in the tissue homogenates were measured using ELISA. All samples were processed in duplicate on a fully automated ELISA system (Human Diagnostics, Germany) and according the manufacturers’ instructions. The used ELISA kits were purchased from Cusabio (Hubei, China) for HSP-90 and COX-2, and R&D systems (Minneapolis, USA) for TGF-β1.

As reported by the manufacturers, the HSP-90α had a detection range between 0.312 to 20 ng/mL with a sensitivity < 0.078 ng/mL, intra-assay precision <8 % and inter-assay precision <10 %. The detection range of the COX-2 kit was 1.56-100 ng/mL, with a sensitivity <0.39 ng/mL, intra-assay precision <8 % and inter-assay precision <10 %. The TGF-β1 kit had a detection range between 31.2-2,000 pg/mL, sensitivity of 4.6 pg/mL, intra-assay and inter-assay precisions of <4 % and <10 %, respectively.

### RNA extraction and cDNA synthesis

Total RNA was isolated from the stored colonic specimens in RNA*Later* following homogenisation of the specimens and by using the Purelink RNA mini kit from Life Technologies (Thermo Fisher Scientific, CA, USA) and according to the manufacturer’s instructions. RNA was treated with RNAse-free DNAse during the extraction protocol to avoid the collection of genomic DNA and the concentrations and quality of the extracted total RNA were measured using the BioSpec-nano (Shimadzu Corporation, Japan), and its quality and integrity were concluded through the A260/A280 ratio.

For cDNA synthesis, 200 ng of total RNA was transcribed to cDNA using a high capacity RNA-to-cDNA Reverse Transcription Kit from Applied Biosystems, (Thermo Fisher Scientific, Warrington, UK) following the manufacturer’s protocol.

### Quantitative RT-PCR

Quantitative RT-PCR was performed using the 2^-∆∆Ct^ method on the following 4 target rat genes: Wnt (NM_001105714.1), β-Catenin (AF397179.1), DKK-1 (NM_001106350.1) and COX-2 (AF233596.1). The results were normalised against the Ct values of β-actin (NM_031144.3) and expressed as fold-change compared with the normal control group. The nucleotides primer sequences are listed in Table 1.

**Table 1:** The sequences of PCR primers used for the detection of β-actin, Wnt, β-catenin, DKK-1 and COX-2 including the corresponding genes accession numbers

	Forwards	Reverse
β-actin (NCBI: NM_031144.3)	5′ CGG TCA GGT CAT CAC TAT CG 3′	5′ TTC CAT ACC CAG GAA GGA AG 3′
Wnt (NCBI: NM_001105714.1)	5′ AGC TGG GTT TCT GCT ACG TT 3′	5′ AAT CTG TCA GCA GGT TCG TG 3′
β-Catenin (NCBI: AF397179.1)	5′ TTC CTG AGC TGA CCA AAC TG 3′	5′ GCA CTA TGG CAG ACA CCA TC 3′
DKK-1 (NCBI: NM_001106350.1)	5′ ATT CCA GCG CTG TTA CTG TG 3′	5′ GAA TTG CTG GTT TGA TGG TG 3′
COX-2 (NCBI: AF233596.1)	5′ AAT CGC TGT ACA AGC AGT GG 3′	5′ GCA GCC ATT TCT TTC TCT CC 3′

PCR reactions were carried out by using power SYBR green master mix from Applied Biosystems, (Thermo Fisher Scientific, Warrington, UK) and a step one Real Time PCR system (Applied Biosystems, USA) in triplicate wells. Each well of the PCR plate contained 10 μl SYBR Green, 7 μl DNase/RNase free water, 1 μl of each primer (5 pmol) and 1 μl cDNA (25 ng). The amplification was performed under the following conditions: 40 cycles (95 °C 15 s and 60 °C 1 min). Two negative controls were included, one with minus-reverse transcription (minus-RT) control from the previous reverse transcription step and a minus-template PCR, which contained all the PCR components but water was used as a template.

### Statistical analysis

Statistical analysis of the results was performed using SPSS version 16. Normality and homogeneity of data were assessed with the Kolmogorov-Smirnov test and Levene test, respectively. One way ANOVA followed by LSD post hoc test were used to compare between the different groups. Data are expressed as mean ± standard deviation and P value < 0.05 was considered significant.

## Results

### Routine biochemistry

There was no significant difference (P > 0.05) between the different study groups in body weight, liver enzymes and renal function parameters (Table 2). However, serum concentrations of total 25-OH Vitamin D were significantly higher in the VitD (P = 0.01 × 10^−4^) and 5-FU/D (P = 0.0002) groups that received cholecalciferol compared with the other study groups (Table 2).

**Table 2:** Mean ± SD of body weight, colon surface area (length × width in cm), count of colonic tumours by gross and dissecting microscope, large ACF, number of tumour/colon surface area ratio (NT/CS), serum 25-OH vitamin D concentrations, liver enzymes and renal function parameters and in the different study groups

		Normal group	AOM group	5-FU group	VitD group	5-FU/D group
Body weight (g)		231.57 ± 20.01	221.97 ± 23.01	238.42 ± 13.64	230.1 ± 22.2	229.5 ± 18.7
Colon surface area (cm^2^)		19.1 ± 2.2	18.89 ± 3.43	19.81 ± 1.84	21.5 ± 2.29	20.4 ± 4.1
Tumour count	Gross	N/A	12.5 ± 3.21	8.7 ± 2.9**	8.1 ± 2.5**	6.2 ± 1.6**^,^***
	Dissecting Microscope	N/A	17.36 ± 4.6	8.8 ± 1.75**	10.04 ± 3.6**	5.78 ± 1.1**^,^***^,^****
	Total	N/A	29.16 ± 2.92	16.66 ± 4.41**	19.33 ± 1.8**	11.5 ± 1.65**^,^***^,^****
large ACF (with ≥ 4 crypts/focus)		0	41.3 ± 8.0	21.3 ± 5.3**	19.7 ± 7.6**	11.2 ± 4.2**^,^***^,^****
NT/CS Ratio		N/A	1.5 ± 0.14	0.83 ± 0.14**	0.86 ± 0.06**	0.6 ± 0.18**^,^***^,^****
25-OH Vitamin D (ng/mL)		46.19 ± 8.1	36.6 ± 6.7*	35.7 ± 9.5*	68.5 ± 9.1*^,^**^,^***	65.8 ± 9.1*^,^**^,^***
ALP (IU/L)		122.6 ± 11.2	125.7 ± 9.7	120.8 ± 12.4	127.3 ± 11.9	121.6 ± 11.1
ALT (U/L)		67 ± 2.4	71.2 ± 6.7	68.7 ± 4.1	66.4 ± 5.3	67.3 ± 3.7
AST (U/L)		92.4 ± 24.2	105.8 ± 26.7	109 ± 21.6	103 ± 19.8	99 ± 11.9
Creatinine (mg/dL)		0.22 ± 0.03	0.2 ± 0.06	0.2 ± 0.03	0.19 ± 0.03	0.21 ± 0.05
Urea (mg/dL)		47.6 ± 5.1	52.3 ± 4	51.6 ± 9.5	47.3 ± 5.8	49.1 ± 3.7
BUN (mg/dL)		22.2 ± 2.4	24.4 ± 1.9	26.3 ± 4.4	22 ± 2.7	21.4 ± 2.1

*P < 0.05 compared with normal; **P < 0.05 compared with AOM group, ***P < 0.05 compared with 5-FU group and ****P < 0.05 compared with VitD group

### Quantification of tumours and histopathological findings

**Fig 1:**
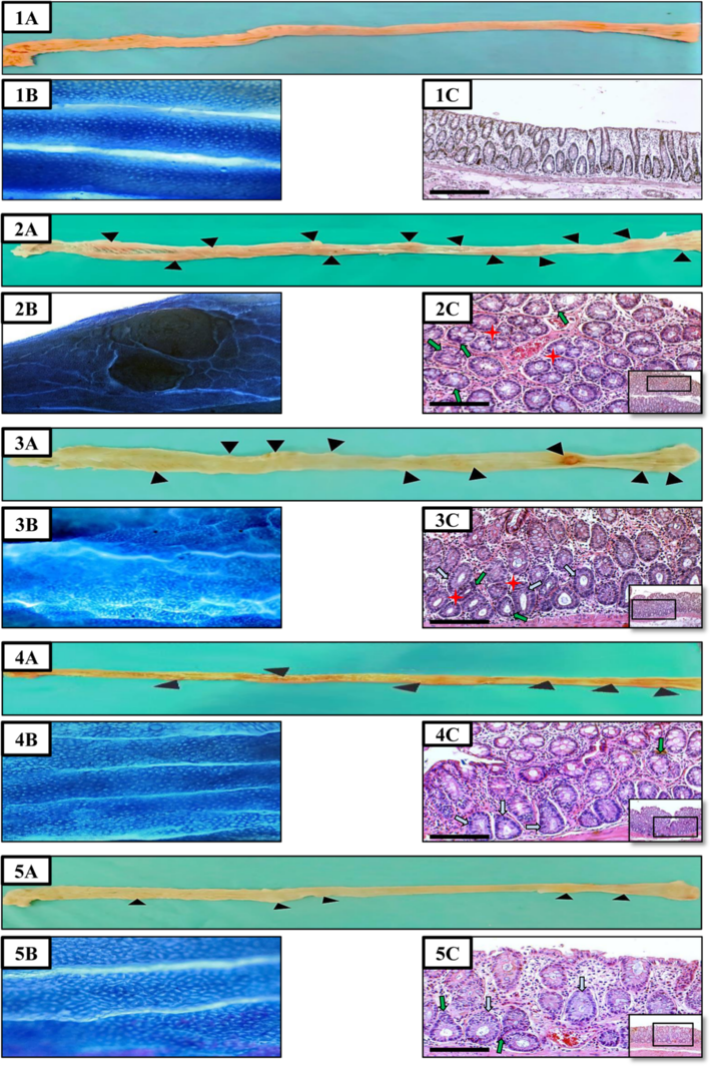
Gross and microscopic appearance of colon mucosa in (1) control group, (2) AOM group, (3) 5-FU group, (4) vitamin D group and (5) 5-FU/D group. Colonic mucosa was examined by (Panel **a**): Gross; (Panel **b**): dissecting microscopy following staining with 0.2 % methylene blue and (Panel **c**): light microscopy at magnifications ×100 and ×200 following staining with H&E. (Black arrow head = tumour observed by naked eye; red star = large ACF [>4 crypts/focus]; light blue arrow = hyperplasia and green arrow = dysplasia)

Administration of AOM had resulted in the formation of tumours on the mucosal surface of all colon specimens collected from the injected groups. However, in rats injected with AOM and then treated with 5-FU, vitamin D, or a combination of both drugs there was a significant decrease in the numbers of grown tumours as detected by gross and dissecting microscope examination (Table 2). The lowest number of tumours was observed in the ‘5-FU/D group’ compared with the other treatment groups (Fig. 1; panel 5a). Furthermore, the number of tumours/colon surface area ratio (NT/CS) was also significantly lower in the ‘5-FU’ (P = 0.02), ‘VitD’ (P = 0.04 × 10^−2^) and ‘5-FU/D’ (P = 0.02 × 10^−5^) groups compared with the ‘AOM group’ (Table 2).

Examination by dissecting microscope following methylene blue staining showed normal mucosal and crypt appearance in the control group (Fig. 1; panel 1b). The AOM group demonstrated several micro-tumours and significant alteration of the mucosal surface (Fig. 1; panel 2b). Treatment with 5-FU significantly decreased the numbers of tumours but the colonic mucosal architecture was distorted (Fig. 1; panel 3b). Similar effect on the numbers of observed tumours under the microscope was also seen in the group treated with vitamin D3 monotherapy. However, the distortion of the mucosal topographic features was less marked compared with the ‘AOM’ and ‘5-FU’ groups (Fig. 1; panel 4b). The combination of vitamin D3 with 5-FU also preserved/restored the mucosal architecture compared with ‘AOM’ and ‘5-FU’ groups (Fig. 1; panel 5b).

Histopathological examination showed the growing of multiple tubular adenomas and the presence of many large ACF (>4 crypt/focus) with hyperplastic and dysplastic glandular epithelium in the colon specimens collected from the ‘AOM group’ (Fig. 1; panel 2c). Only 2 rats of the AOM group developed adenocarcinoma while the remaining study groups were free.

The use of 5-FU alone following AOM injections showed a significant reduction in the numbers of large ACF and the thickness of the colon mucosa compared with AOM group (P = 0.01 × 10^−6^). However, marked hyperplasia and high grade dysplasia of the glandular epithelial cells were observed despite the observed decrease in the quantities of large ACF (Fig. 1; panel 3c). The use of cholecalciferol (vitamin D3) either alone (P = 0.03 × 10^−7^) or in combination with 5-FU (P = 0.02 × 10^−9^) also reduced the numbers of large ACF compared with AOM group and the lowest numbers were observed in the ‘5-FU/D’ group (Table 2). Furthermore, glandular dysplasia was less frequently observed and it was characterised of being of low grade in the groups treated with vitamin D3 compared with the other groups (Fig. 1; panel 4c and 5c). Nevertheless, vitamin D3 did not prevent/restore glandular epithelial hyperplasia in the treated groups.

### Immunohistochemistry findings

**Fig 2:**
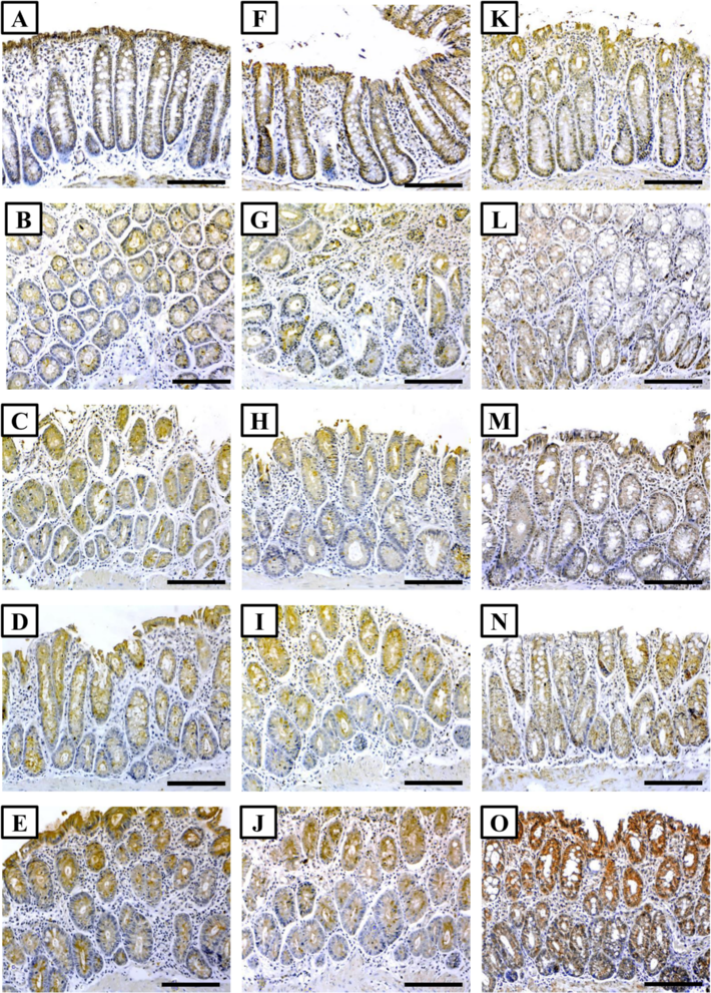
Immunohistochemistry localisation of TGF-β1 (left column), TGF-β type 2 receptor (middle column) and smad4 (right column) in control (**a**, **f** & **k**), AOM (**b**, **g** & **l**), 5-FU (**c**, **h** & **m**), VitD (**d**, **i** & **n**) and 5-FU/D (**e**, **j** & **o**) groups. (×200 magnification, scale bar = 8 μm)

Immunostaining of normal colon specimens obtained from the control group showed that the expression of TGF-β1, its type II receptor and smad4 was localised in the cytoplasm of the glandular epithelium. In the AOM group, a significant decrease in the expression of the three proteins was observed, especially in glands with marked dysplasia (Fig. 2b, g and l). A significant increase in the expression of TGF-β1 and its related molecules was noted following the treatment with 5-FU (P = 0.04 × 10^−3^), vitamin D3 (P = 0.05 × 10^−5^) and 5-FU + vitamin D3 (P = 0.02 × 10^−4^) (Table 3). The highest increase in the expression of the candidate molecules was observed with the ‘5-FU/D’ group compared with the other study groups including control (Table 3).

**Table 3:** Mean ± SD of immunohistochemistry scores for TGF-β1, TGF-β receptor II, Smad 4, β-catenin, iNOS and HSP-90 proteins in colon specimens

	Normal group	AOM group	5-FU group	VitD group	5-FU/D group
TGF-β1	315.5 ± 31.2	136.4 ± 23.4*	301.48 ± 44.3**	323.9 ± 38.5**	348.1 ± 27.8*^,^**^,^***
TGF-β receptor II	333.7 ± 28.7	121.4 ± 19.2*	135.7 ± 17.6*	229.2 ± 26.8*^,^**^,^***	377.2 ± 17.8*^,^**^,^***^,^****
Smad 4	301.1 ± 26	147.2 ± 20.3*	216.1 ± 23.9*^,^**	258.7 ± 31.6*^,^**^,^***	386.3 ± 12.2*^,^**^,^***^,^****
β-catenin	61 ± 16.7	370.6 ± 28.7*	267.6 ± 35.3*^,^**	259.6 ± 30.1*^,^**	149.7 ± 22.6*^,^**^,^***^,^****
iNOS	39.4 ± 9.5	373.1 ± 26.8*	261.8 ± 47.2*^,^**	188.1 ± 33.6*^,^**^,^***	113.1 ± 21.6*^,^**^,^***^,^****
HSP-90	112.7 ± 27.6	376.5 ± 22.6*	302.4 ± 36.8*^,^**	174.7 ± 28.6*^,^**^,^***	88 ± 15.4*^,^**^,^***^,^****

*P < 0.05 compared with normal; **P < 0.05 compared with AOM group, ***P < 0.05 compared with 5-FU group and ****P < 0.05 compared with VitD group

**Fig 3:**
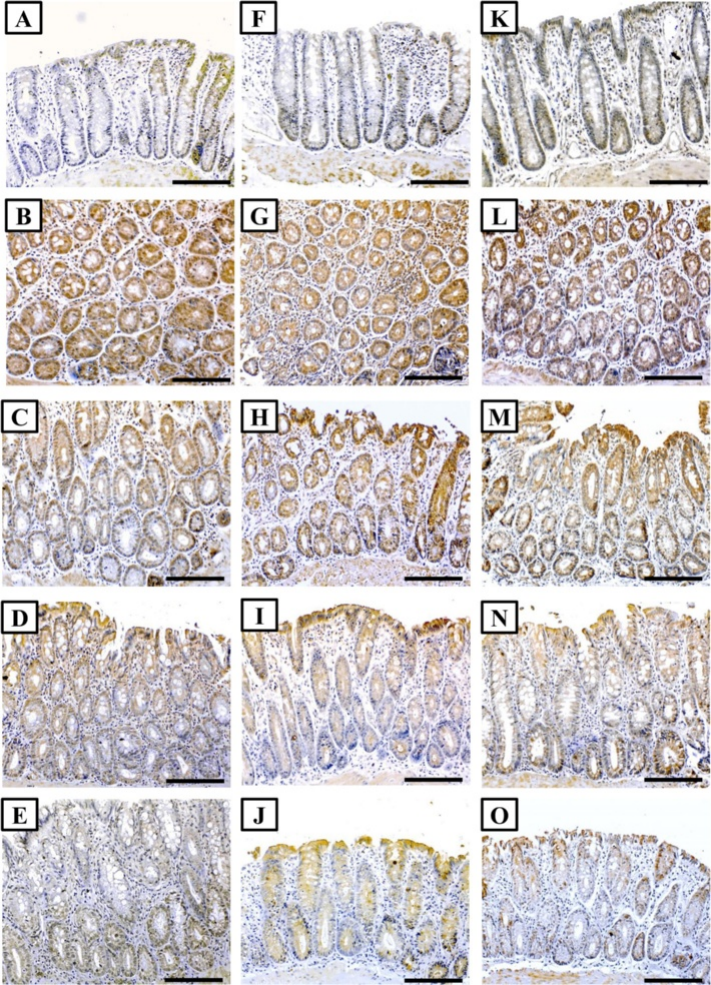
Immunohistochemistry localisation of β-catenin (left column), iNOS (middle column) and HSP-90 (right column) in control (**a**, **f** & **k**), AOM (**b**, **g** & **l**), 5-FU (**c**, **h** & **m**), VitD (**d**, **i** & **n**) and 5-FU/D (**e**, **j** & **o**) groups. (×200 magnification, scale bar = 8 μm)

On the other hand, the immunostaining of β-catenin, iNOS and HSP-90 was weak in the glandular epithelium of normal colon and the expression was scattered and only detected in few glandular cells (Fig. 3a, f and k). A significant increase in the intensity of the immunostain was observed in the ‘AOM’ group for β-catenin (P = 0.01 × 10^−8^), iNOS (P = 0.01 × 10^−5^) and HSP-90 (P = 0.03 × 10^−6^) compared with control. All proteins showed cytoplasmic expression except for β-catenin which exhibited strong nuclear staining (Fig. 3b, g and l). A marked decrease in the expression of β-catenin, iNOS and HSP-90 was seen in the ‘5-FU’ (P = 0.03 × 10^−4^; P = 0.05 × 10^−6^ and P = 0.03 × 10^−5^, respectively), ‘VitD’ (P = 0.002; P = 0.04 × 10^−3^ and P = 0.02 × 10^−6^, respectively) and ‘5-FU/D’ (P = 0.02 × 10^−5^; P = 0.07 × 10^−5^ and P = 0.01 × 10^−8^, respectively) groups when compared with ‘AOM group’ (Table 3). No staining was observed in all negative controls (data not shown).

### ELISA

**Fig 4:**
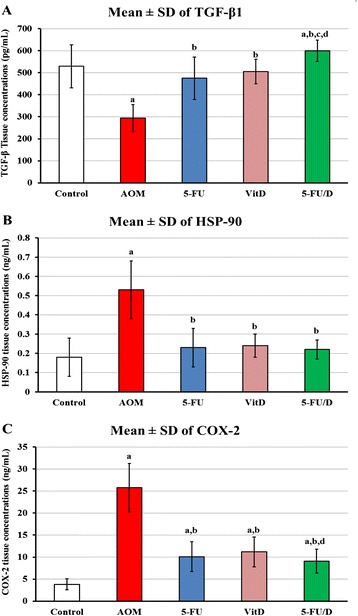
Mean ± SD of tissue homogenate concentrations of (**a**) TGF-β1, (**b**) HSP-90 and (**c**) COX-2 in the different study groups. (a = P < 0.05 compared with control group; b = P < 0.05 compared with AOM group; c = P < 0.05 compared with 5-FU group and d = P < 0.05 compared with VitD group)

The concentrations of TGF-β1 protein in the colon tissue homogenates showed a significant decrease in the ‘AOM group’ (294.7 ± 61.4 pg/mL; P = 0.003) compared with ‘control group’ (529.6 ± 97.8 pg/mL). A significant increase in the protein concentrations was observed in the ‘5-FU’ (475.2 ± 96 pg/mL; P = 0.006 × 10^−3^), ‘VitD’ (505.6 ± 56.3 pg/mL; P = 0.03 × 10^−4^) and ‘5-FU/D’ (600.2 ± 48.7 pg/mL; P = 0.02 × 10^−5^) groups (Fig. 4).

A significant increase in the concentrations of HSP-90α was detected in the tissue homogenates collected from the ‘AOM group’ (0.53 ± 0.15 ng/mL; P = 0.007 × 10^−7^) when compared with those obtained from the ‘control group’ (0.18 ± 0.1 ng/mL). Treatment with 5-FU (0.23 ± 0.1 ng/mL; P = 0.0003), vitamin D (0.24 ± 0.06 ng/mL; P = 0.002) and 5-FU/D (0.22 ± 0.05 ng/mL; P = 0.02 × 10^−4^) was associated with a significant decrease in the protein concentrations when compared with ‘AOM group’. However, there was no significant difference between the three treatment groups (Fig. 4).

The concentrations of COX-2 increased significantly in the ‘AOM’ group (25.8 ± 5.5 ng/mL; P = 0.01 × 10^−5^) compared with the control group (3.83 ± 1.28 ng/mL). Treatment with 5-FU (10.1 ± 3.4 ng/mL; P = 0.0004), vitamin D (11.2 ± 3.4 ng/mL; P = 0.02 × 10^−3^) or combining both drugs (9.1 ± 2.7 ng/mL; P = 0.005 × 10^−3^) significantly reduced the levels of COX-2 in tissue homogenates compared with ‘AOM’ group. However, the levels were still significantly higher than the control group (Fig. 4).

### Quantitative RT-PCR

**Fig 5:**
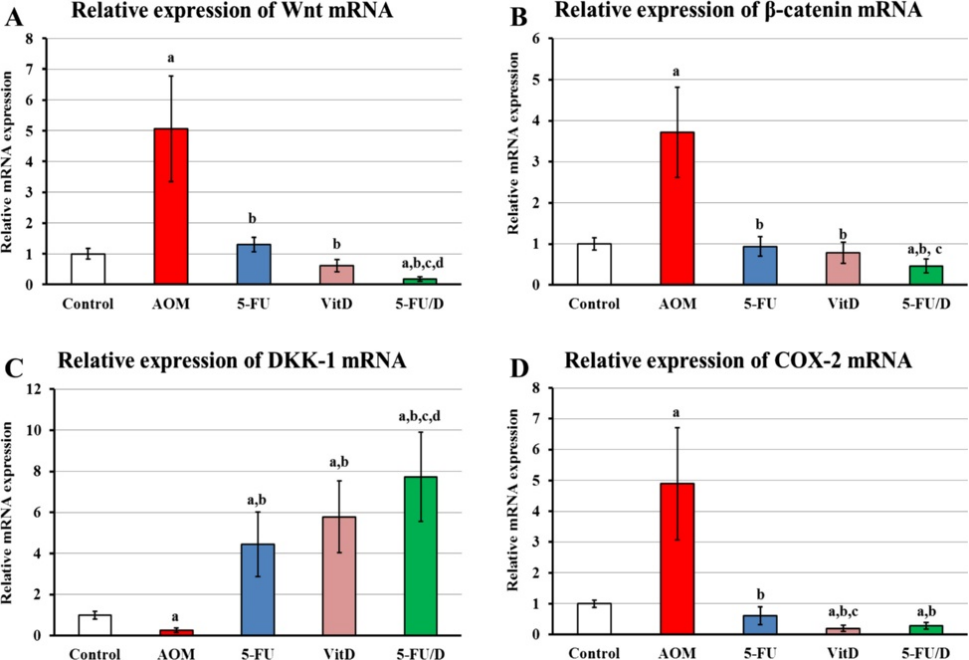
Relative concentration of messenger RNA expression of (**a**) Wnt, (**b**) β-catenin, (**c**) DKK-1 and (**d**) COX-2. (a = P < 0.05 compared with control group; b = P < 0.05 compared with AOM group; c = P < 0.05 compared with 5-FU group and d = P < 0.05 compared with VitD group)

Gene expression study showed a significant increase in the mRNA expression of Wnt (5 folds; P = 0.001), β-catenin (3.8 folds; P = 0.03) and COX-2 (5 folds; P = 0.0002), and a significant decrease in the gene expression of DKK-1 (4 folds; P = 0.0002) in the ‘AOM group’ when compared with the ‘control group’. Treatment either with 5-FU, vitamin D3 or dual therapy significantly altered the gene expression of the aforementioned genes compared with the ‘AOM group’ (Fig. 5).

## Discussion

Herein, we examined the effects of vitamin D3 therapy alone and in combination with 5-FU for the treatment of colon cancer induced in rats by AOM. The current study also measured the possible synergistic interactions between vitamin D3 and 5-FU in the regulation of cell proliferation, differentiation and angiogenesis in colon cancer at the molecular level by measuring the expression patterns of Wnt/β-catenin pathway, TGF-β and its related molecules, HSP-90, COX-2 and iNOS at the protein and gene levels.

Gross and microscopic findings of this study showed that monotherapy with 5-FU, in a similar dosage regimen to that applied for the treatment of CRC in human, significantly reduced the progression of the induced colon cancer in AOM-injected rats. Nevertheless, disruption of the mucosal topography, marked hyperplasia and high grade dysplasia of the glandular epithelium were frequent despite the reduction in the numbers of tumours. At the molecular level, 5-FU increased the expression of TGF-β1 protein, its type 2 receptor and intracellular mediator smad4. Moreover, 5-FU significantly reduced the expression of β-catenin protein and Wnt mRNA, and significantly increased the DKK-1 mRNA expression. The expression of HSP-90, iNOS and COX-2 were also significantly decreased following treatment with 5-FU monotherapy.

Our results further demonstrated that therapy with vitamin D3 had similar effects to 5-FU monotherapy regarding the tumour regression and the expression of candidate molecules. Interestingly, a significant additive anti-tumour effect was obtained from co-therapy with 5-FU and vitamin D3, whereby the most recorded reduction in the numbers of tumours, decrease in the grade of cellular dysplasia and modulation of the candidate pro- and anti-tumorigenesis molecules were observed in the rats treated with 5-FU/vitamin D3 dual therapy. Furthermore, vitamin D3 ± 5-FU preserved the topographic architecture of the colon mucosa and decreased the level of cellular dysplasia but not hyperplasia. Our findings suggest that vitamin D enhances the tumouricidal effects of 5-FU against colon cancer probably by modulating several molecular pathways involved in cell growth, differentiation, DNA damage and angiogenesis.

The importance of vitamin D in the prevention and/or treatment of a variety of cancers, including colon cancer, have been suggested by several studies [39]. The anti-cancer properties of vitamin D can influence the process of proliferation, differentiation and apoptosis of human colon cancer cells mainly through vitamin D receptor (VDR) [40]. This is further supported by the observation that VDR^−/−^ null mice had increased susceptibility to carcinogenesis due to increasing oxidative DNA damage and alteration in the balance between proliferation and apoptosis [41]. The growth of cancer has also been reported to be more aggressive with vitamin D deficiency in a mouse MC-26 model of colon cancer [26] and dietary vitamin D significantly reduced the incidence of colonic tumours in rodents treated with carcinogens [42, 43]. A significant reduction in the number of carcinogens induced tumours has also been shown following the administration of vitamin D3 or several of its analogues [44].

Our results correlate with the aforementioned reports as they showed a significant decrease in the number of colon tumours following treatment with vitamin D. Additionally, vitamin D was associated with less frequent cellular dysplasia of the glandular epithelium. The present findings support the notion that supplementation with vitamin D may decrease and/or reduce the aggressiveness of colon cancer growth and progression.

Despite being a standard anti-CRC chemotherapeutic agent, 5-FU exhibits only limited efficacy when used as a single agent with objective tumour responses from 7-17 % and with a median survival time <1 year [45]. Furthermore, the combination of 5-FU with irinotecan and Leucovorin for the treatment of metastatic CRC cases is also associated with low response rate ranging between 35- 39 % [45]. Thus, there is a compelling need to develop new therapeutic strategies for the treatment of CRC. One possible approach could be by combining natural biological agents that possess anticancer properties to improve the therapeutic efficacy of the currently used anticancer therapy [46].

The combination of vitamin D3 with 5-FU in the present study increased the tumouricidal effects of the cytotoxic drug and it was associated with the lowest numbers of tumours compared either with 5-FU or vitamin D monotherapy. These observations suggest a synergism between both drugs against the progression of colon cancer and this is in agreement with several recently published reports. A research group has lately shown that the cellular sensitivity to chemotherapy increased significantly following supplementation with either vitamin D or its analogues through promoting the synthesis of calcium sensing receptor (CaSR) in vitro [33, 34]. Similar results have also been reported by Milczarek et al. who have demonstrated that the vitamin D analogues, PRI-2191 and PRI-2205, resulted in a significant increase in the expression of VDR and significantly promoted the chemosensitivity of cancer cells to 5-FU therapy in MC38 mouse colon cancer and, HT-29 cell line in vitro and in a xenograft mouse model of colon cancer [22, 35]. Both analogues enhanced the anti-metastatic activity and prolonged the anti-tumour effect of chemotherapy in a dose dependent manner.

These findings, in addition to our observations, indicate a potential beneficial effect of adding vitamin D to chemotherapy for the treatment of CRC. Although promising, the value of the additive anti-tumouricidal effects of vitamin D3 and its analogues with 5-FU still needs further investigations in clinical settings. Furthermore, the observed anti-cancer effects associated with vitamin D could, to some extent, be a result of a positive feedback mechanism on the production of VDR and/or CaSR by colorectal mucosa.

Recently, it has been shown that enterocytes are cable of synthesising active vitamin D independent from the renal system since the colorectal mucosa expresses VDR and vitamin D synthesising (CYP27B1) and catabolising (CYP24A1) enzymes [47–51]. Coherently, several in vivo and in vitro studies have also shown that supplementation with vitamin D3 and its analogues induced a significant increase in the expression of VDR and CaSR by normal and cancerous colorectal mucosa [22, 33–35, 52, 53]. However, an increase in the expression of VDR and vitamin D metabolising enzymes have also been documented in early stages of differentiated CRC [51, 52, 54, 55]. Accordingly, further studies are still required to measure the effect(s) of vitamin D3 ± 5-FU on the expression of vitamin D metabolising enzymes, VDR and CaSR in early and advanced CRC.

Vitamin D has been shown to regulate the signalling mechanisms of several molecular pathways to prevent/treat colon cancer. The development and progression of colon cancer at the molecular level is complex and involves disruption of several molecular pathways that modulate cell growth, differentiation and angiogenesis. Well documented pro-carcinogenic pathways include abnormal increase in the Wnt/β-catenin signals [9–11, 56], HSP-90 [16–18], COX-2 and iNOS by colonic glandular cells [12–15]. Moreover, inhibition of the TGF-β1 signals in these cells through mutations in its type 2 receptor and intracellular mediators is also associated with cancer progression [6–8].

A variety of studies have shown that vitamin D prevents cell proliferation and differentiation by inhibiting the transcriptional activities of Wnt/β-catenin and promoting the expression of TGF-β and its signalling molecules through VDR [41, 57–59]. In this regard, gene knockout of VDR in SW480-ADH cells increased the transcriptional activities of β-catenin and inhibited the suppressive effects of vitamin D on β-catenin target genes [41]. More recently, vitamin D has also been shown to inhibit the expression of Wnt, β-catenin and their target genes in normal colon cells in vitro and in vivo [60]. Vitamin D analogues also inhibit the growth of xenograft models of human colon cancer and this effect was achieved through several mechanisms including increasing the production of DKK-1, a pure inhibitor of Wnt/β-catenin signalling, expression in the xenografts [61, 62]. Similar effects have also been reported in chemically induced model of colon cancer in rodents [63, 64].

Deregulation of the TGF-β1 signalling mechanism is well documented in colon cancer and the cancer cells develop resistance to the cytostatic effect of TGF-β1 by mutations in the TGF-β type 2 receptor and deletion of smad4 to allow cancer progression [6, 7, 65–68]. Although several studies have shown that vitamin D modulates the expression of TGF-β1 and its related molecules in a variety of tissues [69, 70], only a limited number of reports in colon cancer are available. TGF-β has been shown to promote the expression of VDR and to regulate vitamin D signalling pathway through smad3 in vitro [71]. A later study has also demonstrated that treatment with vitamin D up-regulated the expression of TGF-β receptor II through increasing the levels of CaSR in human colonic epithelial CBS cells [72].

Our findings are in parallel with the previous studies as they demonstrate that the observed reduction in the numbers of colonic tumours was associated with a significant decrease in the expression of β-catenin protein, Wnt gene and significant increase in the DKK-1 mRNA, TGF-β1, TGF-β type II receptor and smad4 proteins. Furthermore, the most significant alteration in the expressions of the studied Wnt/β-catenin and TGF-β signalling molecules were detected in the ‘5-FU/D’ group. Taken together, we suggest that the observed synergism between both drugs on cancer cell growth and differentiation could be mediated by several mechanisms including inhibition of the Wnt canonical pathway and promotion of the production of TGF-β signals. However, future studies are required to measure the effects of combining both drugs on the expression of the other molecules involved in the regulation of Wnt canonical (e.g. E-cadherin, TCF) and TGF-β (e.g. Smad2) pathways in vitro and in vivo.

INOS and COX-2 play an important role, among other molecules, in the promotion of colon cancer progression through inducing angiogenesis and DNA damage [73–75]. Experimental studies have also reported that target inhibition of these molecules is associated with less tumour formation and/or regression of cancer [13, 76–78]. Additionally, HSP-90 is essential for cancer progression and it is overexpressed in several tumours including colon cancer [17, 18]. The use of HSP90 inhibitors resulted in the reduction of tumour invasiveness, tumour cell proliferation, vascularisation and improvement in the efficacy of chemotherapy [16].

Currently, the available data in the literature on the effects of vitamin D on the expression of iNOS, COX-2 and HSP-90 in colon cancer is limited. Hence, we hypothesise that vitamin D could promote the efficacy of 5-FU and the regression of colon cancer by downregulating the expression of iNOS, COX-2 and HSP-90 in the colon mucosa to inhibit the processes of angiogenesis and DNA damage. However, more studies are needed to explore the role(s) of vitamin D and its receptor in the regulation of these pathogenic molecules in colon cancer to support our hypothesis.

In conclusion, our results suggest that vitamin D supplementation promotes the efficacy of 5-FU during the treatment of colon cancer through synergistic interactions that modulate cell growth, differentiation, apoptosis, angiogenesis and DNA damage by targeting several pro- and anti-cancerous molecules. These molecular pathways include Wnt/β-catenin pathway, TGF-β1 signals, iNOS, COX-2 and HSP-90. Further studies are required to illustrate the clinical value of vitamin D supplementation during the treatment of colon cancer with 5-FU.
